# Integrated Healthcare Delivery: A Qualitative Research Approach to Identifying and Harmonizing Perspectives of Integrated Neglected Tropical Disease Programs

**DOI:** 10.1371/journal.pntd.0005085

**Published:** 2016-10-24

**Authors:** Arianna Rubin Means, Julie Jacobson, Aryc W. Mosher, Judd L. Walson

**Affiliations:** 1 Department of Global Health, University of Washington, Seattle, Washington, United States of America; 2 Neglected Tropical Diseases, Global Health, Bill & Melinda Gates Foundation, Seattle, Washington, United States of America; 3 Departments of Medicine, Pediatrics, Epidemiology, University of Washington, Seattle, Washington, United States of America; Common Heritage Foundation, NIGERIA

## Abstract

**Background:**

While some evidence supports the beneficial effects of integrating neglected tropical disease (NTD) programs to optimize coverage and reduce costs, there is minimal information regarding when or how to effectively operationalize program integration. The lack of systematic analyses of integration experiences and of integration processes may act as an impediment to achieving more effective NTD programming. We aimed to learn about the experiences of NTD stakeholders and their perceptions of integration.

**Methodology:**

We evaluated differences in the definitions, roles, perceived effectiveness, and implementation experiences of integrated NTD programs among a variety of NTD stakeholder groups, including multilateral organizations, funding partners, implementation partners, national Ministry of Health (MOH) teams, district MOH teams, volunteer rural health workers, and community members participating in NTD campaigns. Semi-structured key informant interviews were conducted. Coding of themes involved a mix of applying in-vivo open coding and *a priori* thematic coding from a start list.

**Findings:**

In total, 41 interviews were conducted. Salient themes varied by stakeholder, however dominant themes on integration included: significant variations in definitions, differential effectiveness of specific integrated NTD activities, community member perceptions of NTD programs, the influence of funders, perceived facilitators, perceived barriers, and the effects of integration on health system strength. In general, stakeholder groups provided unique perspectives, rather than contrarian points of view, on the same topics. The stakeholders identified more advantages to integration than disadvantages, however there are a number of both unique facilitators and challenges to integration from the perspective of each stakeholder group.

**Conclusions:**

Qualitative data suggest several structural, process, and technical opportunities that could be addressed to promote more effective and efficient integrated NTD elimination programs. We highlight a set of ten recommendations that may address stakeholder concerns and perceptions regarding these key opportunities. For example, public health stakeholders should embrace a broader perspective of community-based health needs, including and beyond NTDs, and available platforms for addressing those needs.

## Author Summary

Neglected tropical diseases are a group of parasitic, viral, and bacterial diseases that are often co-endemic in low resource settings. Five of these diseases (lymphatic filariasis, onchocerciasis, schistosomiasis, soil transmitted helminths, and trachoma) are addressed specifically through a method called mass drug administration, where entire at risk populations are targeted with preventative drug treatments. Because of the geographical and interventional overlap between these diseases, many experts recommend program integration as a method for accelerating their control or elimination. However the optimal approaches for operationalizing integrated programing has not been systematically assessed. We undertook a cross sectional qualitative research study with neglected tropical disease stakeholders to understand different stakeholder approaches to and perspectives on program integration. The stakeholders highlighted different definitions of the term “integration”, the differential effectiveness of specific activities when integrated, the influence of integration on community member engagement, the influence of funders on integrated programming, facilitators and barriers to effective integration, and the effects of integration on health system strength. Our analysis suggests that there are three types of integration to consider: structural, process, and technical. We use these categories to make ten recommendations to stakeholders that might be used to improve integrated neglected tropical disease programming moving forward.

## Introduction

A major challenge facing health systems in low-resource settings is the often ad-hoc and vertically silo-ed approach to organizational structure. Many global health leaders are promoting the integration of vertical programs into a shared delivery infrastructure to strengthen the efficiency, effectiveness, and sustainably of health systems and to optimize resources with the goal of promoting equitable and synergistic health improvements [1–3]. However, the term *integration* is not consistently defined and often incorporates a variety of ideas, including continuity of care, inter-organizational relationships, disease management, and others [4]. In addition, a variety of external and internal factors, including local and national politics and complex institutional pressures are also important drivers of, and barriers to, successful integration [5]. Understanding facilitators and barriers of integration within existing integrated programs offers the opportunity to improve public health integration efforts globally.

The neglected tropical diseases (NTDs) are an example of a group of 17 diseases in which synergistic integration may be ideal given their high degree of geographic and population overlap. Furthermore, five of the world’s most prevalent NTDs are generally considered “tool ready” and are primarily controlled through mass drug administration (MDA), including: lymphatic filariasis (LF), onchocerciasis, schistosomiasis, soil transmitted helminths (STH), and trachoma [6]. Schistosomiasis and STH MDA programs are typically delivered to pre-school and school-age children via school-based delivery platforms. LF, onchocerciasis, and trachoma programs, on the other hand, are typically delivered via community-based delivery platforms. LF and onchocerciasis MDA programs are uniquely delivered using community directed intervention (CDI) strategies.

Triple drug administration (TDA) is one option for integrated treatment through the simultaneous provision of albendazole, ivermectin, and praziquantel or azithromycin. Studies suggest that TDA is clinically effective and cost-efficient, and the WHO recommends TDA of albendazole, ivermectin and praziquantel in areas that have had one to two previous rounds of treatment [8, 11, 18]. However, TDA is not widely utilized in co-endemic countries, even those with established integrated NTD programs. In addition to preventative treatment strategies such as MDA there are a number of other activities that can potentially be integrated between NTD programs. NTDs that are co-endemic in the same geographic area can integrate activities such as mapping, vector control, community sensitization and education, health worker training, surveillance, monitoring and evaluation, and disability management.

In January 2012 an array of partners gathered to endorse the World Health Organization (WHO) NTD Roadmap and launch the London Declaration, a commitment to pursuing control or focal elimination of select NTDs by 2020 [7]. Integration of NTD programs is considered to be one of the most promising approaches for achieving the London Declaration goals, and integrated NTD programming has been endorsed and recommended by the WHO for NTD endemic countries to optimize program implementation [8, 9]. However, while there is evidence suggesting beneficial effects of integration on NTD program coverage [10] and costs [11], there is negligible information regarding best practices in operationalizing integration. Similarly there has been minimal examination of the potential detrimental impacts of integration.” The lack of a systematic analysis of integration experiences and complexity limits the ability of programs to optimize NTD program implementation in co-endemic areas [12]. Given that integrated NTD programs are now encouraged in endemic countries, there is currently a unique opportunity to learn from stakeholders as they engage in integrated programming.

In this study we aimed to identify how perceptions regarding the role, effectiveness, and implementation of integrated NTD programs differ among various NTD stakeholders. We also aimed to describe program integration and best practices for implementing integrated programs from each stakeholder’s perspective. We focus specifically on the NTDs for which MDA is the standard of care due to geographic and interventional congruencies, as well as to limit the scope of this analysis.

## Methods

### Study design and conceptual model

**Fig 1:**
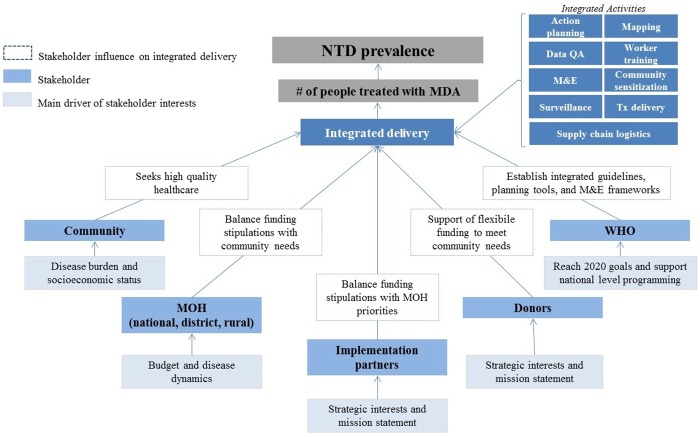
Theoretical model of NTD integration stakeholders, simplified stakeholder interest drivers, and influence on integrated delivery.

We conducted a qualitative cross-sectional study to identify and harmonize NTD stakeholder approaches to integrated program delivery. We identified seven primary stakeholder groups involved in and affected by integrated programming of NTDs, including partners at multilateral organizations, funding partners, implementation partners, national Ministry of Health (MOH) teams, districts MOH teams, volunteer rural health workers (known as community drug distributors, CDDs), and community members participating in MDA campaigns. The conceptual model in Fig 1 outlines the stakeholders involved in integrated NTD programs as well as simplified descriptions of their driving interests and influences on NTD integration.

### Sampling frame and recruitment strategy

The sampling methodology utilized the NTD stakeholder group (N = 7) as the unit of analysis, as determined through a maximum variation approach. An overarching tenet of maximum variation is the understanding that each stakeholder group must be considered separately, and thus a distinct sampling frame and sampling strategy was identified for each group.

The sampling frame for stakeholders working in multilateral organizations and funder organizations was individuals deemed as subject matter experts (i.e. key informants). In this study, subject matter experts are defined as influential individuals recognized in the NTD domain as thought-leaders or policy-influencers, who often present and publish in the field. Subject matter experts must have been working in the field of NTDs for ten or more years.

The sampling frame for the implementation partners and national MOH workers was limited to subject matter experts working in five highly co-endemic countries (i.e. endemic to all five MDA-indicated NTDs) and thus these stakeholders work in a variety of NTD-endemic countries throughout sub-Saharan Africa and Asia. The sampling frame for district and rural MOH workers was even further limited to one country of in-depth focus. The country of in-depth focus was selected because it is endemic for all five NTDs for which MDA is the standard of care, noted above. It is located in sub-Saharan Africa, and the population is largely rural. The name of the country cannot be provided, in accordance with Institutional Review Board (IRB) stipulations.

The recruitment strategy used to identify all stakeholders—with the exception of community members—was purposive quota sampling of mutually exclusive key informant groups [13]. The strategy was deemed appropriate as this research aims to capture and equally value the range of NTD stakeholder perspectives, from influential key informants at the global level to program implementers at the local level. Only individuals working on two or more of the five NTDs for which MDA is the standard of care were recruited.

For community members, the sampling strategy was random purposive sampling. During four community-based MDA campaigns in the country of focus, community members gathered to receive NTD health education and treatment at schools and health posts. Education was provided prior to treatment, and then treatment was delivered to community members throughout the day. During the campaigns, a translator or CDD made an announcement that community members may be approached and invited to participate in interviews regarding their experiences with NTD programs. The translator or CDD was instructed to approach every 5^th^ woman who participated in MDA treatment, starting with the first woman treated. This probabilistic sampling strategy for community members was used as this group is much larger than the other stakeholder groups and we aimed to capture a more representative sample of these NTD stakeholders.

Due to the large number of individual stakeholders in each stakeholder group, it was not possible to ensure complete data saturation.

### Interview structure

Semi-structured key informant interviews with a mix of respondent and informant style questions were used. Participants were asked both to describe the process through which they engage in integrated programs as well as their recommendations to others regarding how integration should be pursued. They were asked to explain their rationale for these recommendations, describe factors that facilitate success, and describe barriers that challenge successful integration. Many of the questions were similar across stakeholders in order to compare and contrast answers. However, questions were also specific to the experiences and roles of the particular stakeholder.

### Ethics

All interviews were audio recorded and participants were required to provide verbal consent prior to the commencement of the interview. This methodological approach was granted exemption status from the University of Washington IRB committee under a minimal risk determination status. Exemption was granted with the understanding that no country names or personal identifiers of interviewees would be available.

### Data Management and Analysis

Transcripts were uploaded to the software program Atlas ti. (V.7 2012). Coding of key themes involved a mix of applying in-vivo open coding and *a priori* thematic coding from a start list [14].

After the first round of coding was completed, a second round was conducted using an adaptation of the constant comparative coding method. Constant comparative methods help conceptualize and describe the variety of responses in the data [15]. This set of codes and memos were used to identify themes that highlight common trends across responses [14]. In qualitative research, themes are recurrent unifying concepts or statements about the topic of inquiry [16]. Within each theme group, we identified responses that were similar, opposing, or unique to each stakeholder group. By undertaking this two stage analysis process we utilized a mix of case-oriented and variable-oriented analytical strategies [17].

## Results

### Study participants

A total of 41 interviews were conducted with stakeholders: 2 from multilateral organizations, 2 from funding partner agencies, 4 implementation partners, 5 national MOH health workers, 6 MOH district health workers, 8 CDDs, and 14 community members. Salient themes varied by stakeholder however dominant themes arising during analysis were relevant to (1) variations in definitions of “integration”, (2) differential effectiveness of integration according to the specific NTD activities integrated (3) community perceptions of integrated NTD programs, (4) the influence of funders on NTD integration, (5) perceived facilitators of integration, (6) perceived barriers to integration, and (7) the effect of integration on health system strength. Within each of these themes, we identified where feedback was similar, differed, or was unique to each stakeholder group. In the section that follows these themes are discussed in greater detail, with a selection of supportive quotes provided in context.

### Theme 1: There are variations in definitions of the term “integration”

All participants, with the exception of community members, were asked to provide a definition of the term “integration” within the context of NTD programs. In general, definitions were broad, even within stakeholder groups, and participants often provided rationales for integration rather than a working definition. Health workers, particularly CDDs, described integration of specific activities while individuals at multilateral, funder, and implementing organizations focused on definitions of integration relevant to upstream planning and measurement.

Integration is doing several activities at one time in one day. For example, distribution of Mectizan together with the salt testing for iodine. We can also be measuring the growth of under 5 children at the same time, giving us more efficiency

–CDD 5

The idea of integration is not to have a disease focused approach but to think about the program outcomes to accomplish, what must be done, and how and for whom [necessary activities] overlap

–Multilateral 1

A number of participants noted the lack of an existing definition but cautioned against being too definitive, citing the need to maintain flexibility in applications of the term.

Seeing some of the conversations people *don’t* have, I don't think there’s a standard [definition]. On some of these things, the minute we define them or put parameters around it, you restrict yourself instantly. And that’s a problem

–Funder 1

Conversely, several stakeholders noted the danger of having such ubiquitous, vague terminology.

I think integration, you know, has got this buzz. “Integration!”, but it means different things to different people at different times in different ways and to really define it, we have to be very, very specific. It becomes a buzz word and then people didn’t want to hear it anymore for a while. “Don’t say integration. Say coordination”. It’s hard, but I think we just need to be much more defined about what we mean here

–Implementation Partner (IMP) 4

The lack of consensus regarding the definition of integration was evident in the many ways stakeholders used the term to describe various clinical, political, and organizational processes. However, the most common definition of integration across stakeholders was the act of coordinating specific activities and interventions when mutually relevant to two or more health programs. Some stakeholders also identified integration as a way to transfer knowledge between programs, particularly for diseases at different stages of elimination.

### Theme 2: Integration is differentially desirable and effective, depending upon the specific NTD activities under consideration

**Fig 2:**
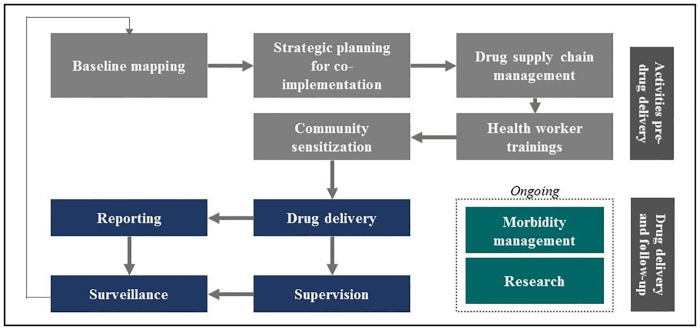
Summary of activities required for delivery of mass drug administration campaigns.

Multiple stakeholders argued that there was a common misconception that integrating NTD programs for the five MDA-indicated diseases simply requires co-delivering all designated drugs. Rather, there are a number of activities involved in NTD control in addition to drug delivery (Fig 2). These activities can be integrated with one another or with complementary co-interventions such as safe water, sanitation, or nutritional interventions. But stakeholders unanimously noted that not all of these activities are contextually or scientifically appropriate for integration across NTDs.

It depends on the activities which can be integrated…There are some activities which you can totally not integrate. So we shouldn’t force matters, to say, “Let’s integrate. Let’s integrate,” but let’s look at the activities. Can we integrate these activities? Can we co-implement these products?

–MOH National 2

It’s not integration for its own sake. It’s smart program implementation and management

–Funder 1

All of the specific program activities mentioned by stakeholders during interviews are discussed below.

#### Baseline mapping

Most MOH and implementation stakeholders were skeptical regarding the efficiency of integrated mapping efforts due to the need to modify existing mapping technologies and methodologies. Implementation stakeholders in particular noted that there are a number of approaches to developing integrated sampling strategies for mapping and subsequent surveillance, all of which would need to be considered carefully from both epidemiological and operational perspectives.

I think you could integrate everything together on the same [mapping] survey if you get all the right people together and they all agree on the sampling framework and collection methods. But I'm more concerned about whether the field teams can actually do that… what is the capacity of the team and how much time, extra time, they would spend out in the field to collect additional specimens during integrated surveys?

–IMP3

#### Program planning

Stakeholder feedback indicated that integrated planning is a crucial and often overlooked aspect of integrated programming. Stakeholders mentioned that often integrated programs are launched without careful epidemiological mapping, considering disease specific targets, program maturity, integration history, or thinking through the specific activities that should be integrated or remain vertical across programs.

…we need to get our plan straight about how we are going to do this and we need to make sure that we know what we want and get consensus within our community…otherwise it can get really complex really quickly…it can stifle progress for a while

–IMP4

Each country is in a different stage of absorbing [integration] and taking it on. And I think one of the mistakes that people make is you say okay, we write up a national plan and we say we’re going to integrate… It does not happen overnight. And it phases in, and I don't think people have thought about that

–Funder1

During discussions on planning, most stakeholders mentioned NTD Steering Committees. Implementation partners in particular notes that a critical aspect of planning is empowering an NTD Steering Committee that serves as an entity for harmonizing partner and MOH interests into a broad and long-term country-specific implementation strategy.

If [partners] are not coordinated well before implementation you create huge tensions between partners in the field and that is not good, that's not helpful to any program. It is really important for partners to have those conversations to work out how are we going to work together, how are we going to support the ministry, and then go in with a well-coordinated integrated strategy

–IMP3

#### Supply chains

National MOH stakeholders highlighted supply chain integration as an important activity in successfully integrated programs. If supply chains are not appropriately coordinated it can delay co-distribution of drugs to the community. Various MOH stakeholders reported that these delays can undermine long-term program credibility and community desire to engage in integrated programming if drug delivery is repeatedly postponed. However, multilateral partners noted difficulties with integrating supply chains due to the different ways in which drugs are shipped and managed.

Another challenge with integration is supply chains…Ivermectin is already in the country, ready for MDA but they just put it by the doors waiting to go to the community because albendazole is not yet here

–MOH District 6

#### Health worker training

Health worker training was one of the activities that national MOH stakeholders were most enthusiastic to integrate. They noted that the degree to which trainings should be integrated depends upon which level of health provider is targeted (for example, CDDs versus district-level environmental health technicians). For CDDs, some MOH stakeholders cautioned against consolidating two disease-specific trainings into one integrated training. Rather they recommended that the same number of integrated trainings take place whereby individual CDDs learn about the same diseases multiple times. No recommendations were made regarding specific training tools for integrated trainings. In contrast, CDDs recommended that they participate in fewer vertical trainings overall, particularly trainings that are repetitive and uncompensated.

#### Community sensitization

Most MOH stakeholders said that integrated community sensitization activities have the benefit of presenting a coherent message to community members, both across NTD programs and other community-based delivery programs. The MOH stakeholders reported that integration of community member education is less controversial relative to CDD education, because the messages are simpler and effectiveness is highly influenced by consistency.

If we use those resources together and get whatever is our target population together, possibly the effectiveness will be much—I want to believe much, much higher than it could if we wanted to do them independently. And the other disadvantages of doing that independently is that when you go into the community, you find that you may be telling them the same information, but from just a slightly different angle. They’re going to say, “But this, we have already heard this thing. Why are they coming again like this?”

–MOH National 2

#### Drug delivery

Drug delivery was one of the most contentious activities to integrate, with stakeholders exhibiting particular hesitancy around potential programmatic transitions to triple drug administration TDA-based MDA programs. Several MOH stakeholders in favor of TDA were of the opinion that drug co-delivery would facilitate greater community participation in MDA activities. Some CDDs also explained that co-delivery regimens such as TDA would give them more time for farming, from which they generate more income than they currently do from the allocated program incentives.

I think more people would participate when you are using one stop to kill several bits. They would come in large numbers because we will not bother them a lot. We just go to the community and say, “Okay. This year we’re going to do MDAs for schisto, for LF, for oncho, for STH at one go.’ After doing that, we are done with them. Otherwise, they get tired of the participation

–MOH District 6

We are needing to go into the fields, so if those drugs were together we would have only one day to treat two types of diseases so it saves time we can spend outside in the fields

–CDD2

A number of stakeholders mentioned that co-delivery of drugs or TDA in particular will only be feasible with improved community sensitization efforts and political will. A number of additional challenges associated with TDA are described in sections below.

#### Supervision

MOH stakeholders described that there are multiple layers of supervision required in NTD programs: national MOH workers supervise district workers, district workers supervise local health workers and community activities, etc. District MOH workers reported that integrated supervision of community activities is extremely effective. Integrated supervision saves them time and two district workers reported that it also improves their relationship with the communities in which they work.

Doing supervision of the community should be integrated. If you go and come back for all these things, each time separately, people say that, “this person doesn’t know how to leave. She comes here quite often just to check on one thing and she goes—the thing you are interested in is something else, other than the job you are doing.” So if you integrate you go and you check all the disease registers, you discuss everything and then you come back so you give time to those people to reflect on other things

–MOH District 5

#### Reporting

Individuals across stakeholder groups said that MOH reporting activities are not typically integrated. MOH health workers often found this paradoxical given that they are at times completing disease-specific reporting forms for integrated activities targeting multiple NTDs simultaneously. MOH stakeholders discussed complex disease reporting procedures as an impediment to conducting integrated data quality assurance, monitoring, and evaluation.

Implementation and multilateral partners pointed to recent efforts to develop integrated reporting forms to addresses these issues. However, a number of MOH stakeholders highlighted that these forms are not yet user friendly. Additionally, multilateral stakeholders pointed out that some rich disease-specific information is lost in integrated reporting forms.

Depending on the country, you see very complex forms that try to integrate everything together. Who is filling these out? It's mostly farmers who don't have a lot of education and we're not making it easy for them. And so if that's the key entry for data and it's already being entered incorrectly and then it's just being aggregated up, what kind of quality data are we getting?

- IMP3

Integrating forms adds efficiency in reporting by creating one form but by trying to improve the efficiency we may have decreased quality…There is less information now for expert panels to review and give advice on. It dilutes the quality of [disease-specific] guidance that can be provided. At the same time without it, we couldn’t see the whole country as a whole picture

- Multilateral 2

#### Morbidity management

Morbidity management of individuals with advanced NTD sequelae was an activity that the majority of stakeholders reported should remain vertical. They cited that while the identification of individuals in need of morbidity management may easily be integrated into MDA or mapping activities, the overlap in target populations requiring surgery for specific diseases may not be extensive. One stakeholder described how there are two models for integrating morbidity management with other program activities. In one model, programs attain cost-effectiveness by integrating resource intensive activities such as identifying people in need of surgery, seconding experts from other areas, and transporting patients to specialty treatment areas. In another less efficient model, these activities are referred to as integrated, but are actually conducted serially, one after the other. None of the stakeholders discussed integrating morbidity management into the health system more broadly.

#### Research

Implementation partners and funders similarly noted that integrated research drives integrated implementation. However several of the implementation partners highlighted that preemptive vertical priority setting is a necessary step to avoid compromising scientific rigor when trying to integrate research efforts, such as developing integrated sampling methodologies or new diagnostics; thus disease specific end points can at times drive integrated methodological innovations. Additionally they described how research that promotes standardized integrated NTD metrics, methods, and delivery systems across NTDs as well as other diseases is crucial for building the evidence base necessary for achieving comprehensive universal health coverage.

Research is very complex because each NTD has…its own life cycle, its own specific dynamics, ecological dynamics and transmission dynamics, etc. So sometimes one size will fit all and sometimes it won’t and we have to figure out when it does fit all and when it doesn’t, when it makes sense and when it doesn’t. Having more people at the table with more perspectives is good. You develop more innovative and creative methods. But sometimes it’s hard, too, because it takes longer to get there and you have to compromise to satisfy more parties

–IMP4

Standardized [data] collection methods are essential to create opportunities for an integrated approach whether it’s the immediate NTDs that we're focused on or new opportunities

–IMP3

### Theme 3: Community members have favorable perceptions of NTD programs, but few explicit opinions regarding integration

Community members had unique feedback regarding the acceptability and feasibility of integrated NTD programs. Amongst those interviewed there was unanimous fear of NTD-associated morbidities, particularly elephantitis and visible worms in stool. This fear translated to strong community demand for MDA programs.

When those volunteers come with those medicines, we receive it with a green light because we know that those medicine which they are coming with, they are one, for free. Two, they give hope for life. Once you have been attacked by these diseases, you cannot even dress well because the leg swells so badly

–Community Member (CM) 15

There were three points of constructive feedback from community members regarding MDA in general. First, during pre-treatment community sensitization and education it is often not clear which diseases are being treated simultaneously or during different MDA treatment rounds. Second, MDA programs can demand considerable time from community members to participate in all education, registration, and treatment activities. Depending upon the treatment schedule, these NTD activities can occur several times per year and are likely one of many community-based health campaigns in which community members are asked to participate. Lastly, some community members desired more treatments, particularly expanding praziquantel treatment to adults in schistosomiasis endemic areas. These points of feedback were echoed by volunteer CDDs.

When we do [MDA] in different days what happens is the people may not understand. They’re going to think that the drug which has been given at the second day is the same drug which was given in the first, then they will miss out on one of the treatments

–CDD5

When we talk of primary healthcare, it’s integrating all the health programs together and bringing them to the community. And with that the community then knows and gets used to it. But we have brought everything vertical, everything alone. And now when you come to the community, they will even tell you "I’m confused". Yesterday you came and told me that my nutrition is this, this, and this. And yet today you are coming and saying my nutrition is this—which one now should I follow?” Each program, they want to achieve their own goals with their own messages… So maybe working together we would have also relieved these confusions which we bring to the community

–CDD4

Women noted that men in particular are likely to be absent from MDA events when they perceive that the campaigns require a large amount of time throughout the year for each separate program.

It’s hard for these male people because they need to find food for the family. So, they can’t force themselves to stay there and wait for medicine while at home there is no food. That’s why the [CDDs] don’t find these males

–CM6

In general it was difficult for community members to discuss their preferences regarding integrated activities relative to vertical activities. When asked, one community member shrugged and said, “This is government decision, when they say to receive drugs, we just accept it, and we don’t have an option.” (CM9). When asked about their potential participation in integrated treatments in the future, some community members feared taking too many tablets at once during integrated campaigns. Additionally, two community members mentioned concerns that further integration would actually make treatment days longer, especially if there are only a few health workers present at each campaign.

If you would say four, five tablets, I say oooh no, four or five tablets at once. That must be too heavy. We’d say no, from fear

–CM3

However, the majority of community members independently noted that they are accustomed to taking several medications at once, repeatedly providing the example of malaria treatment regimens and pain killers. Most of those in favor of integrated MDA drug delivery focused on the time savings that would be accrued.

If you take both type of medicines together, you prevent both types of diseases, while if you take one type of medicine, then you might see that other disease… when you take both types of medicine at once, you still have time to do other things. That means your time is your time

–CM14

Several district and rural health workers noted that community members are unknowingly already accustomed to integrated NTD programs through co-delivery of schistosomiasis and STH services in schools and LF and onchocerciasis programs in communities. One CDD was surprised that some might consider programs such as LF and onchocerciasis separately in the first place, “From our side, we only call it the oncho program…though I suppose we know the filariasis is there” (CDD 2). Similarly, although several programs have successfully integrated activities in some co-endemic countries, it is clear that integrated programming has led to some confusion on the part of CDDs and, as a result, community members. One CDD erroneously explained, “We combine treatments because Mectizan is there to prevent disease while albendazole is there to cure worms that are already too much so it’s good to combine them. One is for prevention and the other is for cure for which is already there” (CDD3).

Many community members cited the expanded program on immunizations (EPI) as an example for why integrated community-based programs are acceptable and advisable. Community members noted that “first clinic” (EPI and growth monitoring during child health weeks) was appropriately integrated because all activities shared the common goal of promoting child growth. These community perceptions were echoed by district workers in the same communities.

Let’s take it from the way we evolved from doing child health days. Previously we were giving under-fives so many different programs, but now you find that we’ve brought several other activities together. So that kind of integration—that helps people to get all services under one roof and then it improves the turnout

–MOH District 2

### Theme 4: There are a number of multi-level factors that stakeholders perceive as integration facilitators

Interview participants identified four main facilitating factors of integration including the need for efficiency with limited resources, strong central leadership, conception and launching of new integrated programs, and continued relevancy post elimination. The need for program efficiency was the most frequently cited driver for and benefit of integration during stakeholder interviews. Specifically, MOH stakeholders at every level described financial and time efficiencies as the primary factors encouraging program integration in low income settings. For example, several national MOH health workers noted that when they don’t have adequate funding to hold a CDD training on one disease, they will utilize resources earmarked for another disease to provide an integrated training.

Without integration there would only be funding for one training rather than four trainings. So even in delivering drugs, it’s a matter of just bringing those drugs in one vehicle and distributing them rather than coming separately for each disease, where, at the end, you are putting much pressure to the [District Health Office]. At the end, we would not be able to assist

–MOH District 6

Almost all of the stakeholders interviewed, with the exception of community members, stated that integrated strategies will be most effective if they are first institutionalized at the national level of a health system before being launched at district or local levels.

The LF coordinator comes and says, “Okay. I want the volunteers to do this and this.” And off he goes. Next time the onchocerciasis coordinator comes and says, "Okay, I want to meet you guys" and, starts again telling us or teaching us about [onchocerciasis]. Then next time, oh, we get tired… Integration should be started from the national level. With so many coordinators at the national and district level you are trying to create the programs vertically

–MOH District 4

Most multilateral, implementation, and MOH stakeholders said integration at the national level is most efficient if there is a single NTD coordinator overseeing disease-specific NTD focal persons. Such a leader can promote cross-disease coordination and improved communication at the national level which, according to MOH stakeholders, will trickle down to the peripheries.

Each disease has its own unique needs and you still need to have someone behind each of those diseases…But, having an individual who can take responsibility for the larger NTDs, I mean that's desirable in terms of integration, having someone with the big picture, especially when you have specific activities that can be integrated

–IMP3

The country should begin by sensitizing the program managers, knowing that like in Africa, the resources, they'll never be enough so they need to come together, sit together as program managers, work together, share. If people in districts see that you are working together, they will work together as well

–MOH National 3

Additionally, several multilateral and donor partners remarked that it is easier to launch new integrated programs in a country than it is to integrate extant disease-specific programs due to entrenched institutional identities. One multilateral stakeholder noted that, in this capacity, NTD Steering Committees endowed with decision making power (not simply endorsing entities) are critical in the integration process to ensure that Integrated NTD Master Plans are available with actionable recommendations and integrated activities.

What we are doing is very very simple. Providing the drugs is simple. Record keeping is simple. Of course reaching people can be complex. But newly integrated programs combine efforts and it becomes even more simple… It is very rare that a country with a Steering Committee would decide not launch programs as integrated. It is just logical

–Multilateral 1

Additionally, as countries work towards NTD elimination, some MOH stakeholders noted there are political incentives for national and sub-national health workers to integrate their workloads and programs. Specifically, it allows programs and personnel to have continued relevancy and expertise after single diseases have been locally eliminated.

### Theme 5: There are a number of multi-level factors that stakeholders perceive as integration barriers

Stakeholders identified four primary barriers to effective NTD program integration: (1) a fear amongst MOH workers that they will lose their jobs or recognition of their work, (2) external timelines or funder pressures that do not allow for a lengthy integration processes, (3) tensions between school and community-based delivery proponents, and (4) the fact that some strong or well-funded programs do not see integration as a “win-win”. These barriers are driven by political and administrative factors.

Stakeholders at all levels reported that integration is often not pursued or is inefficiently pursued when stakeholders perceive that they won’t be able to maintain some degree of disease-specific autonomy and recognition of program-specific markers of success.

The other thing that could be a challenge in integration is basically the attitude of those people who manage programs asked to integrate. Attitude, prestige, come in and begin to affect the way we work. Some people want to take all of the glory, all of the success, all of the compliments that come, so they would feel like if they bring in other people, they may lose that kind of a degree of complementarity that goes to them. That now would have to become a shared success

–MOH District 2

It has been in the ministry’s interest to have integration, for the sake of time and money, but what I see is it’s maybe us individuals that are the problem…we have been wanting vertical programs because coordinating together, it’s like I’m going to lose my powers

–IMP1

While leveraging strong programs to increase the coverage or efficiency of weaker programs is a primary rationale for integration, several stakeholders expressed concern that integrating strong and weak programs could be to the detriment of strong programs. However, when asked, stakeholders could not provide examples of a situation where this phenomena had occurred.

If one program manager is laissez-faire, the laissez-faire will affect the whole integrated thing. Now, this other person that was hardworking would feel like they would lose a little bit success…If somebody’s scoring usually 80 percent. You’ll find once you’ve integrated with someone scoring 75, now you’re scoring 65

–MOH District 3

Several district level MOH workers and implementing partners also expressed concerns that successful integrated programming might be used as a rationale for minimizing the financial support provided to sub-national disease control teams.

Often one car is used to supply everybody. It’s like: “Well our malaria car broke down, but thank God the schistosomiasis car is coming in.” Then it’s like, “but now you only get one car because malaria and schisto—they’re working together.” Well, if you were managing things from the perspective of playing off all the vertical programs to get your resources, does that sound like a good deal? It sounds like a good deal for the people who buy the cars

–IMP2

MOH stakeholders unanimously noted that vertical funding mechanisms limit the ability for NTD programs to integrate at national and sub-national levels. Some stakeholders recommended that funders and implementation partners take more responsibility for ensuring greater collaboration between disease-focused groups while other stakeholders recommended that governments take more responsibility for ensuring coordination amongst NTD partners working in a country. Funder influence is discussed in greater detail in *Theme 6*.

I think it could be fiefdoms, disease-specific groups who look at the disease-specific track but don’t look at how it relates to the other diseases. Some groups come in and say, “we just look at our piece and we’re not gonna worry about what the platform is to get all the diseases moving forward.” And so you kind of just work in your silo

–Funder 1

My advice is to the government because whenever each NGO comes in the country, they have to sign a memorandum of understanding with the government and it is the government which has to assign that NGO to a particular place… And I know that each NGO comes with its own goals and what have you. But the government can still say, “Okay, you are doing part A and we have an NGO which is doing part B. Is it possible that you should work together?”

–IMP1

Another identified barrier to achieving more extensive NTD program integration is the operational and political divides between community-based MDA programs (LF, onchocerciasis, and trachoma programs) and school-based MDA programs (schistosomiasis and STH programs). For example, according to several implementation and multilateral stakeholders TDA has not been introduced or scaled-up in any country largely due to the political paradigm shift that would need to take place prior to providing treatments to children in community-based settings.

And I think it has to do with politics. It has to do with the fact that there are school-based people and there are community-based people. And when you start linking everything into community-based, which you have to for triple drug administration, the school-based people are unhappy. So in the great political compromise, it’s kept as the status quo: you pay 40% more and things are kept as two distinct rounds [of treatment] so that the school-aged treatment advocates are not threatened

–IMP2

Many of the community members recommended that all treatment take place during community campaigns so that parents can supervise their children. Without advocating for one delivery system over another, the majority of implementation partners and MOH stakeholders commented on the political complexity of choosing either school or community based delivery over the other. Some national stakeholders argued that more school-age children will miss integrated treatments if targeted in the community, while a number of CDDs argued that integrated community-based treatments could expand treatment to children who do not attend school. Several stakeholders noted that shared responsibility between Ministries of Education and Health means that there is an authority vacuum where both entities have a stake but neither claims ownership for the disease program.

When the community-based [treatments] aren’t needed anymore, then you just switch over to the school-based… The idea is to find the people who are suited to provide the treatment…A large argument is related to cost. And another large argument is related to sustainability. Not enough is said about penetrance to the poor who may not be able to pay school fees and be there. And gender is a major issue in a lot of places where I work, because girls are less likely to go to school

–IMP2

Lastly, many stakeholders highlighted that while integration is necessary and desirable in some locations, stakeholders should still track disease specific outcomes. A lack of disease-specific outcomes limits funder or partner ability to track impact and may diminish their desire to pursue integrated programs. Specifically implementation partners and multilateral stakeholders recommended disease-specific coverage metrics as indicators of integrated program success.

The idea is you go about implementing programs together in a way that results in efficiencies and cost savings but does not compromise the specific objectives of any of the disease-specific programs that are being integrated

–IMP2

What are we aiming to do? We aren’t aiming to integrate, we are aiming to eliminate a disease. You can quantify how much a program is integrated, but you have to monitor the progress of specific disease indicators

–Multilateral 2

Similarly, although stakeholders noted opportunities for NTDs to integrate with other community-based health programs within a comprehensive primary healthcare system, this broad integration is often not undertaken because stronger, well-funded programs often do not see integration with NTDs as a “win-win”.

When discussing integration between NTDs and tuberculosis there was a comment “um, well, we can do that as long as it doesn’t affect our program in any way”…and I said, “I mean if you integrate, it’s going to change the dynamic and it could have an impact on your [progress and goals], but maybe for a broader good ultimately”. But it was sort of like, I’ll integrate, but I don’t want any of our programs to be affected in any way and I was like, “okay, maybe then you don’t really want to integrate.”

–IMP4

### Theme 6: Funders have a profound influence on NTD integration activities

Many MOH stakeholders cited that integrated programs would be more effective if funder and partner resources were more easily integrated. A common theme arising in this regard was related to incentives for volunteer CDDs; given that programs rely on a largely volunteer workforce, the sustainability of the programs is compromised by changes in volunteer incentivizing. MOH stakeholders recommended that community volunteer incentive schedules align across NTDs. They also recommended that NTD incentives align with other disease programs, noting that well-funded programs such as HIV may compromise less-funded programs such as NTDs by offering larger incentives to health workers for similar work.

Now in the community you will find organizations giving CDDs incentives which is far much better than what we give. And our job is only done once, maybe twice. So they feel like this is something of less value…And when there is something that is not of good value in terms of appreciating the effort that you’re putting in, people feel like it is something that is not to be taken seriously

–MOH National 2

You have to look at sustainability. Because if funders just come with a lot of money, they can say $50.00 a day for CDDs, and they'll be very happy. But after three years, the funder will go and that will be the death of the program. These programs that are there, they have to be assimilated in the government system. The funders, they come and go…When they go, still, the government should function

–MOH National 3

And you start looking at all the other programs, not just an integrated NTD program, but you're looking at malaria, you're looking at HIV, you're looking at immunization, nutrition…Some programs provide per diems, others don't, and there are different incentives that are given to volunteers from each program…They don't feel like they're part of anything consistently. And I think we've really sort of failed in that regard at keeping them engaged throughout the year

–IMP3

According to the stakeholders interviewed, funders may unintentionally thwart integration due to their desire for limited resources to be spent efficiently, causing different funders to cover different geographies of a country in a piece-meal manner. Additionally, because some funders only provide resources for specific diseases their resources may not be available for integrated programs. As one stakeholder points out, there is a delicate balance that a funding organization must maintain to ensure that it has fidelity to its mission statement while ensuring maximum health impact.

If donors will only work on one disease then there is no hope that integration will take place. Usually donors just take each part of a country to cover and do some different things. While if they really worked together it would be just one intervention that they work together for training, reporting, such things. The donors can be very convincing and have a lot of power over what happens in the community

–Multilateral 1

In all fairness to the funders, if they're going in and saying we are supporting trichiasis surgeries, then they just want to have information on trichiasis, not eye health overall. So it's like you know, some flexibility is nice not to be so closed off of those opportunities, but I totally understand to a certain extent, like yeah we could quickly start going in another direction, lose focus, and start spending a lot more money than we wanted

–IMP3

However most of the funder, multilateral, and implementation partner stakeholders remarked that funder and implementation partner culture appears to be changing, with increased emphasis on country needs and integrated programming. Newly established NTD coordinating committees might be a facilitating factor.

I think it’s starting to change with some of the partners. I appreciate that organizations have disease-specific mandates. But I think new groups coming in need to be brought into that culture that’s been developed in the existing community. Yes, you may have a disease-specific focus that your institution requires. But on the ground, if we all work together, maybe we can all leverage more bang for our buck… I have always been really impressed by the flexibility but also the positive, informal nature of the NTD community.

–Funder 2

We don’t want to duplicate any efforts. So if you go into a country and you say these three groups are in there supporting the Ministry of Health and they covered everything but trachoma, then we might go in to bring the trachoma piece in. And you could say in a more traditional sense, we’re just supporting trachoma in that country but we really see it being done within the context of an integrative program

–Funder 1

### Theme 7: NTD integration affects NTD program strength, but also has broader health system effects

Stakeholders reported that NTD integration can affect NTD delivery systems as well as the health-system more generally. NTD-specific effects include transferring knowledge from successful programs to scaling programs and strengthening the presence of NTDs on the global health agenda. Broader health system effects include general improvements in community based healthcare programs, encouraging a culture of learning from other disease delivery platforms, and progress towards universal primary healthcare coverage.

A number of stakeholders across levels recommended that in order to maintain NTD scale-up success, integration be pursued in a “fluid manner” (IMP3) based on the maturation of different disease programs and disease dynamics at a local level.

Integration helped improve the coverage of the weaker program in [our country] because we relied on the existing strategies of the stronger program to implement the weaker one…LF was the stronger program at the time so onchocerciasis was added on to it logically and financially and LF is still going on as it should

–MOH National 5

In this regard, stakeholders repeatedly provided LF and STH as examples of programs for which integration might facilitate a transfer of knowledge within the NTD system; geographies that have successfully controlled LF and are transitioning to post-MDA surveillance while attempting to scale-up nascent STH programs could integrate activities to avoid losing progress made by LF programs. These disease programs have particular co-dependencies and a natural opportunity for synergy due to the fact that they utilize MDA of the drug albendazole.

Now the really important piece of integration is about transferring…If we’ve got these programs like trachoma, onchocerciasis, and LF that are going to be shutting down, schistosomiasis and STH are these nascent programs that don’t know what they’re trying to do…But I’m afraid it may wind up, in the end, with one structure collapsing and another having to be build up again

–IMP2

We’re ready to scale up [STH programs]…We need to make sure that there’s really not a hiatus after LF programs are stopped….because you do have the potential to lose some gains if you drop back to nothing

–Donor 2

Several stakeholders also discussed how integration helped NTDs develop a larger presence on global health agendas and include a larger number of stakeholders. Various stakeholders noted that the benefit of integration to NTDs in terms of advocacy and fundraising has been indispensable.

There was some smart rethinking of some major people in the field who looked at the burden and looked at the funding and knew that we had this rapid impact package. We could do a lot for a little bit of money, for less than 50 cents per person at least from a preventive chemotherapy standpoint, and decided to really re-package it and advocate for it in this new framework. So being able to show that the NTDs as a whole has this huge disability…equal to in many cases tuberculosis or malaria or HIV where there’s been a lot more funding, you’re able to address them at very little cost…I think that was very smart and it certainly has had many benefits to leveraging more money and funding into the NTD space. We’re a larger voice together than we are separate and I think it has been beneficial, but certainly challenging in some ways as well

–IMP4

According to a number of MOH stakeholders, NTD program integration could also result in broad health system improvements if it encourages health worker efficiencies. These efficiencies could improve community participation in community-based healthcare activities generally.

Each of us has demands on our own time. If we become more efficient, everybody in the system will have time for spare…If we’re telling the people that we want to meet you at the dissemination and community sensitization on Mectizan, if you bring in the LF, you bring in praziquantel, that could reduce the community’s absence in doing their other works, which will possibly increase the attendance for NTDs and also for other health programs rather than saying, 'today you want them for praziquantel and then next week you'd want them for Mectizan. And then the other week we want them for child health days. People, say “Ah, are we not going to be doing our own things?”

–MOH District 2

Multilateral and implementation partners noted that the act of implementing integrated health programs forces public health workers to learn from what works, to look to programs and platforms that have had success, and to think about what can be learned from them. Several stakeholders argued that a focus on learning from integrated programs will benefit both NTDs and country health systems more broadly.

There's a lot of different platforms out there that are doing great work for other [disease] programs. We're just not aware of all those platforms…And boiling down complex problems into specific problems, identifying what we really want to understand and test with a platform is not easy but it could likely be worth it

–IMP3

A subset of stakeholders across levels also mentioned that establishing an integrated community-based NTD platform affords the opportunity to provide preventative healthcare for NTDs and other conditions outside of formal treatment settings. This is important for achieving universal health coverage of preventative healthcare services and for disease elimination efforts in particular.

Take Ebola for example. When people talk about a health system, they’re talking about the ability to have outreach. And outreach using community-based approaches is part of the health system…They’ve activated a part of the healthcare system that’s a very important part…So what we have to learn from NTDs is how to get out of the healthcare system and find people who are not “sick” yet

–IMP2

So often [community-based] campaigns strengthen the larger health system… You can't just sit back and say, well, the primary healthcare system's gonna figure this all out. By having these opportunities and platforms from a community-based elimination program you do strengthen the capacity of the health system at a very local level, outside of the health units, to do disease surveillance, to do specific activities that I think the primary healthcare system fails at. We're more developed in healthcare delivery, and we often fail in primary healthcare outcomes because of that

–IMP3

Now all the NTDs are enmeshed, because we’re working together in a lot of ways and we’re dependent on each other now. If we reach these elimination benchmarks in this integrated way it will be a proof of principle…It’s a whole new paradigm for how you can potentially eliminate in this bundled integrated fashion

–IMP4

## Discussion

In this qualitative study conducted among NTD stakeholder groups, seven main themes emerged regarding integration. These themes reveal a number of perceptions, facilitating factors, and barriers to integrating NTD programs. In general, MDA programs provide a well-accepted platform for reaching at-risk community members with preventative health services in low-income communities. The primary rationale provided for integrating NTD programs was to build upon existing program strengths for the benefit of one or more programs, achieving financial and human resource efficiencies in the process. However, as highlighted by varying stakeholder definitions of “integration”, the term has often been simplified to mean co-implementation and stakeholders unanimously highlighted that co-implementation only makes sense for certain advantageous activities. Activities that most multilateral, funder, implementation partners, and MOH partners highlighted as particularly advantageous to integrate included planning, health worker trainings, supply chain management, drug delivery, and community outreach. Quotes and opinions provided by stakeholders must be considered within the context of stakeholder positionality within the NTD community. For example, many of the remarks from implementation partners regarded understanding and meeting expectations of target communities and funders.

Stakeholders identified a number of factors facilitating or challenging NTD integration, a summary of which can be found in Table 1. These factors build upon important themes such as community perceptions of integrated NTD programs and funder influences on programs. Stakeholders identified integration barriers similar to those identified in research on integrated primary healthcare in high income countries, including vague visions of change and the absence of guiding operational definitions, amongst others [19]. Another commonality with findings from healthcare programs in higher income settings include that stakeholders in this study acknowledged the essentiality of relationships, trust, buy-in, cooperation and communication for successful program integration [20]. Lastly, a prevailing health systems message was that as NTD elimination goals are met in some countries it is important to leverage the established community-based infrastructure for other NTDs or broader preventative healthcare programs.

**Table 1:** Summary of facilitators and barriers to effective NTD integration, as reported by study participants.

Stakeholder	Integration Facilitators	Integration Barriers
All stakeholders	Efficiencies in time and human resources Increased uptake in services through integrated programming Ability to share elimination lessons learned across disease initiatives Leadership structures that promote communication between disease focal persons	Vague and varying “integration” terminology External timelines or funder pressures that don’t allow for a lengthy integration process Some strong or well-funded programs do not see integration as a “win-win” Political encampments of stakeholders who work on school-based versus community-based NTD programming
Multilateral partners	Communication between disease-specific working groups	Loss of important disease-specific data resulting from integrating and simplifying data collection forms
Funders	Disease specific outcomes that can be quantitatively improved following integration	Difficulty in measuring progress of integrated investments Concern for maintained effectiveness of stronger programs if integrating with weaker programs
Implementation partners	Launching newly integrated programs as opposed to supporting existing disease specific programs	Difficulty in integrating efforts with other partners Absence of some integrated tools and methods, limiting ability to perform some technical integrated activities (ex. mapping)
MOH-national	Need to maintain relevancy after disease-specific elimination goals are met Efficiency with minimal financial resources Strong NTD Steering Committees with decision making capacity Detailed NTD Master Plans with specific actionable integrated activities	Human resource challenges/ fear of unemployment or loss of recognition Vertical funding which prohibits integrated activities Vertical supply chains that can delay treatment Fear of reducing effectiveness of a successful program following integration
MOH-district	Human resource efficiencies Desire to promote streamlined community-based activities Integrated leadership at the national level	Vertical direction and supervision at the national level Fear of losing funding/resources following integration
Volunteer rural health workers	Efficiencies in income generating time expenditure Coordinated trainings that promote unified messaging Perceived increase in community participation	Incentives that discourage concentrated NTD labor inputs relative to other disease programs Confusion in NTD knowledge base
Community members	Efficiencies in income generating time expenditure Presence and acceptability of integrated community programs such as EPI Demand for MDA services that don’t consume excess time Unified NTD messages during community sensitization	Confusion during community sensitization activities Fear amongst some of taking large amounts of medication simultaneously

Based on these findings, we suggest a need for three types of integration to be considered separately and transparently: structural integration, process integration, and technical integration. Structural integration refers to coordination of organizational and human resource arrangements across disease programs. Process integration refers to co-implementation of existing programmatic activities and procedures. Technical integration refers to methodological innovations that can take place across disease programs to introduce or improve co-delivery of services. We also propose a set of ten specific recommendations for improving the efficiency, effectiveness, or acceptability of integrated NTD programs, a summary of which can be found in Table 2. Many of these recommendations echo actions that are already considered best practices in NTD programs, but draw attention to the need to fully enact or strengthen these actions for the purposes of successful and effective integrated implementation.

**Table 2:** Ten Integration Recommendations.

	Recommendation	Rationale provided by stakeholders
*Structural integration recommendations*		
*1*	Establish a single NTD Coordinator for all NTDs for which MDA is the standard of care.	The NTD coordinator could efficiently oversee disease-specific program managers, with an integrated perspective and necessary competencies.
2	Country-level NTD Steering Committees should be established or strengthened where already present.	Steering Committees should review long-term integrated Master Plans that must include detailed planning regarding specific activities that will be integrated and how they may be uniquely assessed for impact.
*Process integration recommendations*		
3	The NTD Steering Committee in each country should establish contextual definitions and rationales for integration.	Rationales for integration should include evidence or hypotheses relevant that will build scientific and administrative consensus and promote a harmonized approach to program delivery.
4	Funders and implementation partners should empower NTD Steering Committees.	Partners must ensure that they are working closely with government institutions and Steering Committees to ensure funds and activities are complementary.
5	Integrated activities and systems should start at the national level of the MOH.	Integrated activities must be institutionalized at the national level to promote the necessary multi-level inter-organizational and inter-professional environment at district and local levels.
6	NTD public health practitioners should ensure that integrated programs communicate clear unified goal to community members	Community members should be made fully aware of what diseases they are receiving treatment for and why. This may involve changing the structure of current CDD training curriculum.
*Technical integration recommendations*		
7	Public health stakeholders should embrace a broader perspective of community-based health needs.	There is much to learn and gain from coordinating with other disease platforms. Additionally, platforms such as EPI, water and sanitation programs, and nutritional interventions provide complementary opportunities for providing preventative primary healthcare.
8	MOHs should incorporate TDA into drug delivery schedules.	TDA may result in greater coverage, time, and resource efficiencies. Promoting TDA will require more specific guidelines and bridging the political divide between school and community-based treatment approaches.
9	Incentives and support systems for community volunteers should be aligned across community-based disease programs.	Integrated approaches to volunteer recruitment and maintenance may results in greater sustained engagement overall.
10	Subnational reporting frameworks should be standardized or redesigned to capture information regarding which NTD program activities are integrated with other activities.	Current data collections methods are confusing for health workers and supervisors working on integrated programs, and aggregated field data do not provide information regarding the effectiveness of specific integrated activities.

### Structural integration recommendations

**Recommendation 1:** Countries may wish to establish a single NTD Coordinator for all NTDs for which MDA is the standard of care. Stakeholders recommended that this NTD coordinator could oversee disease-specific program managers and resource allocation, with frequent communication with the NTD Steering Committee. A leader with an integrated perspective and relevant competencies appears critical in contexts with changing vocabulary and foci, and can help facilitate alignment amongst stakeholders within a given health system [21].

**Recommendation 2:** Country-level NTD Steering Committees should be established or strengthened where already present. Weak committees should be strengthened by increasing decision making capacity and requiring implementation partners to present to the Committee prior to program launchings. In this way Steering Committees could also help coordinate donor funding or support MOH program managers working with multiple donors. Stakeholder theory suggests that meeting the multiple needs of stakeholders on Steering Committees would maximize overall systems effectiveness [22]. Steering Committees should help develop or review long-term integrated Master Plans that must include detailed planning regarding specific activities that will be integrated, how they will be integrated, and how integrated activities may be uniquely assessed for impact. According to study stakeholders, most existing integrated Master Plans do not meet these criteria.

### Process integration recommendations

**Recommendation 3:** Healthcare integration in resource-limited settings is facing a similar definitional challenge that integrated care implementers have faced in higher income countries for decades [4]. Thus the NTD Steering Committee in each country should establish contextual definitions and rationales for integration. Rationales for integration should include evidence or hypotheses relevant to (1) scientific rationales for integration, (2) administrative rationales for integration, and (3) health system rationales for integration. According to strategic change management theory, articulating a shared need for change with consideration of diverse stakeholder roles positively influences implementation effectiveness and structural change efforts [20]. This process will provide a standardized manner for the Steering Committee to consider why they are pursuing integration and any potential unintended consequences. These rationales should be shared with all other levels of the MOH and Ministries of Education, where relevant.

**Recommendation 4:** Funders and implementation partners should empower and work with NTD Steering Committees by coordinating closely with the Committee and ensuring that MOH priorities are paramount [23]. Evidence suggests that funder conditions can hamper resource allocation decisions following strategic planning or produce additional workload for health workers in the field [24]. Thus partners must also ensure that they are working closely with other institutions and organizations to ensure funds and activities are complementary. Contributing to a single integrated Master Plan may be a way to promote inter-organizational coordination from the onset.

**Recommendation 5:** NTD integration is complicated and, given that many of the challenges to effective integration are procedural and behavioral rather than scientifically based, change management activities should be undertaken at the national level and used to clearly communicate integration definitions, rationales, and Master Plan strategies to peripheral district and local MOH offices. While evidence from other studies found topdown management of NTD programs to result in negative feedback from peripheral levels [23], according to district-level stakeholders in this study, a strong core management structure is necessary to promote sustainable consensus on integrated NTD programming. There is a general understanding amongst stakeholders that effective integration is facilitated by having a collective vision, shared strategies, and common culture [25]. A number of change management strategies such as promoting a shared mental model of integrated care, for example, would create an inter-organizational and inter-professional environment necessary for delivery of integrated care [20].

**Recommendation 6:** Many community members cited EPI as an appropriately integrated program because all distinct health interventions clearly share the common goal of promoting child health. This suggests that, in order to promote program acceptability, it is up to NTD public health practitioners to ensure that integrated programs have a clearly expressed unified goal and that this goal is being communicated to the public. In communicating this unified message, community members should be made fully aware of what diseases they are receiving treatment for and why. This may involve changing the structure of current CDD training curriculum and prioritizing community sensitization efforts.

### Technical integration recommendations

**Recommendation 7:** The “justification for integrated delivery systems is to meet patients’ needs rather than providers” [26]. Public health stakeholders should embrace a broader perspective of community-based health needs and available platforms for addressing those needs. For example, most district health workers recommended that child health weeks, which are well attended and accepted, be expanded to include other community-based healthcare delivery activities, including MDA delivery. This will necessitate some technical adjustments to standard clinical procedures. Other evaluations of community-based programs such as EPI have also concluded that shared platforms may have broad health system benefits [27]. Ultimately multisectoral integration between activities such as nutritional intervention, water, sanitation, and MDA will result in more effective programs and thus shorter necessary durations of treatment [28]. However, broader approaches to community-based delivery must be designed carefully in fragile health systems so as not to induce operational problems affecting program quality [23].

**Recommendation 8:** Where appropriate, MOHs should incorporate TDA into drug delivery schedules. Most community members described the implementation method as hypothetically acceptable and other health programs, such as polio immunization initiatives, have similarly identified community dissatisfaction when the number of intervention rounds are high [29]. Yet many health workers do not know that TDA of albendazole, ivermectin, and praziquantel is approved by the WHO for the simultaneous treatment of onchocerciasis, LF, STH, and schistosomiasis [8]. According to stakeholders, promoting TDA will require more specific guidelines from the WHO as well as more deliberate attempts to bridge the political divide between school and community-based treatment approaches. These efforts will require multi-year advanced planning to sync previously vertical NTD programs within an integrated platform.

**Recommendation 9:** Incentives and support systems for community volunteers and health workers should be aligned across NTDs and other community-based disease programs. This will require cooperation by multiple funding partners. By integrating approaches to volunteer recruitment and maintenance there may be greater sustained engagement overall. Such necessary activities would also align with the WHO-endorsed Joint Commitment to Harmonized Partner Action for Community Health Workers and Frontline Health Workers [30]. This recommendation could also be considered a part of process integration.

**Recommendation 10:** Stakeholders should standardize and redesign subnational reporting systems to capture information regarding which NTD program activities are integrated with other activities. Stakeholders reported that current data collection methods are confusing for health workers and supervisors working on integrated programs, and aggregated field data do not provide information regarding the effectiveness of specific integrated activities. Such data are necessary for linking particular integrated interventions to programmatic outcomes or health impacts in non-experimental settings. These data must be shared promptly and transparently with the WHO to ensure global disease control benchmarks are accurately monitored.

### Limitations

One limitation of this analysis is that on one-on-one interviews of community members and health workers took place in private rooms within clinics or in places of work. Responses may have been biased if interviewed individuals felt that their feedback may reach employers or community leaders. A second limitation is that the data analysis did not involve multiple coders and thus intercoder reliability was not possible to establish. Lastly, the stakeholders’ views and opinions reflect a subset of the NTD community and are not representative of all stakeholders. While patterns emerged, complete data saturation by stakeholder group was not sought, nor achieved. Further geographically-specific research should be conducted prior to the introduction of any relevant policy changes.

### Conclusion

Application of a social science approach allowed us to provide a theoretical understanding of a number of similarities and differences between different stakeholder perceptions of the complex process of NTD integration. In general, there was greater variation between groups than within and stakeholder groups provided unique perspectives, rather than contrarian points of view, on the same topics. The stakeholders identified more advantages to integration than disadvantages, however there are a number of both unique facilitators and challenges to integration from the perspective of each stakeholder group. These findings provide both explanatory as well as meditative information to NTD integration stakeholders. The ten recommendations provided draw from the qualitative data to highlight structural, process, and technical opportunities to maximize stakeholder interests while promoting more effective and efficient integrated NTD elimination programs.

## References

[1] Kim J, Farmer P, Porter M. Redefining global health-care delivery. Lancet. 2013;382(9897):1060–9. 10.1016/S0140-6736(13)61047-8 23697823

[2] Binagwaho A, Nutt CT, Mutabazi V, Karema C, Nsanzimana S, Gasana M, et al Shared learning in an interconnected world: innovations to advance global health equity. Global Health. 2013;9(37). 10.1186/1744-8603-9-37 PMC3765795 24119388

[3] Gyapong JO, Gyapong M, Yellu N, Anakwah K, Amofah G, Bockarie M, et al Integration of control of neglected tropical diseases into health-care systems: challenges and opportunities. Lancet. 2010;375(9709):160–5. 10.1016/S0140-6736(09)61249-6 20109893

[4] Kodner DL. All together now: a conceptual exploration of integrated care. Healthc Q. 2010;13 Spec No:6–15. 10.12927/hcq.2009.21091 20057243

[5] Burns LR, Pauly MV. Integrated delivery networks: a detour on the road to integrated health care? Health Aff (Millwood). 2002;21(4):128–43. 12117123 10.1377/hlthaff.21.4.128

[6] WHO. Global Programme to Eliminate Lymphatic Filariasis: Progress Report, 2014. Wkly Epidemiol Rec. 2015;90(38):489–504. 26387149

[7] Hotez PJ. NTDs V.2.0: "blue marble health"—neglected tropical disease control and elimination in a shifting health policy landscape. PLoS Negl Trop Dis. 2013;7(11):e2570 10.1371/journal.pntd.0002570 24278496 PMC3836998

[8] WHO. Rolling out and scaling up integrated preventive chemotherapy for selected neglected tropical diseases. Geneva: World Health Organization, 2013. 23620908

[9] WHO. Success in controlling neglected tropical diseases. Geneva; 2013 http://www.who.int/neglected_diseases/WHA_66_second_day/en/.

[10] Hooper PJ, Zoerhoff KL, Kyelem D, Chu B, Flueckiger RM, Bamani S, et al The effects of integration on financing and coverage of neglected tropical disease programs. Am J Trop Med Hyg. 2013;89(3):407–10. 10.4269/ajtmh.13-0018 23836563 PMC3771274

[11] Evans D, McFarland D, Adamani W, Eigege A, Miri E, Schulz J, et al Cost-effectiveness of triple drug administration (TDA) with praziquantel, ivermectin and albendazole for the prevention of neglected tropical diseases in Nigeria. Ann Trop Med Parasitol. 2011;105(8):537–47. 10.1179/2047773211Y.0000000010 22325813 PMC4089800

[12] Grépin K, Reich M. Conceptualizing integration: a framework for analysis applied to neglected tropical disease control partnerships. PLoS Negl Trop Dis. 2008;2(4):e174 10.1371/journal.pntd.0000174 18446203 PMC2321017

[13] Bernard HR, Ryan GW. Analyzing qualitative data: Systematic approaches. Thousand Oaks, CA: SAGE publications; 2009.

[14] Corbin J, Strauss A. Basics of qualitative research: Techniques and procedures for developing grounded theory. Thousand Oaks, CA: Sage Publications; 2008.

[15] Boeije H. A purposeful approach to the constant comparative method in the analysis of qualitative interviews. Quality & Quantity. 2002;36:391–409.

[16] Boyatzis R. Transforming Qualitative Information: Thematic and Code Development. Thousand Oaks, CA: Sage Publications; 1998.

[17] Miles MB, Huberman AM. Qualitative Data Analysis: A Methods Sourcebook. 3rd ed Thousand Oaks, CA: Sage Publications; 2014.

[18] Coulibaly YI, Dicko I, Keita M, Keita MM, Doumbia M, Daou A, et al A cluster randomized study of the safety of integrated treatment of trachoma and lymphatic filariasis in children and adults in Sikasso, Mali. PLoS Negl Trop Dis. 2013;7(5). 10.1371/journal.pntd.0002221 PMC3649960 23675549

[19] Suter E, Oelke ND, Adair CE, Armitage GD. Ten key principles for successful health systems integration. Healthc Q. 2010;13 Spec No:16–23. 20057244 10.12927/hcq.2009.21092 PMC3004930

[20] Evans JM, Baker GR. Shared mental models of integrated care: aligning multiple stakeholder perspectives. J Health Organ Manag. 2012;26:713–36. 10.1108/14777261211276989 23252323

[21] Viktoria Stein K, Rieder A. Integrated care at the crossroads-defining the way forward. Int J Integr Care. 2009;9:e10 19513179 PMC2691940

[22] Brugha R, Varvasovszky Z. Stakeholder analysis: a review. Health Policy Plan. 2000;15(3):239–46. 11012397 10.1093/heapol/15.3.239

[23] Cavalli A, Bamba SI, Traore MN, Boelaert M, Coulibaly Y, Polman K, et al Interactions between Global Health Initiatives and country health systems: the case of a neglected tropical diseases control program in Mali. PLoS Negl Trop Dis. 2010;4(8):e798 10.1371/journal.pntd.0000798 20808908 PMC2923152

[24] Global Health Partnerships: Assessing Country Consequences. Bill and Melinda Gates Foundation and McKinsey & Company. 2005. http://www.who.int/healthsystems/gf16.pdf.

[25] Schwartz JI, Dunkle A, Akiteng AR, Birabwa-Male D, Kagimu R, Mondo CK, et al Towards reframing health service delivery in Uganda: the Uganda Initiative for Integrated Management of Non-Communicable Diseases. Glob Health Action. 2015;8:26537 10.3402/gha.v8.26537 25563451 PMC4292588

[26] Rogers A, Sheaff R. Formal and informal systems of primary healthcare in an integrated system: evidence from the United Kingdom. Healthc Pap. 2003;1(2):47–58; discussion 104–7. 10.12927/hcpap.2000.17218 12811065

[27] Anand A, Luman ET, O'Connor PM. Building on success—potential to improve coverage of multiple health interventions through integrated delivery with routine childhood vaccination. J Infect Dis. 2012;205 Suppl 1:S28–39. 10.1093/infdis/jir794 22315383

[28] Gazzinelli A, Correa-Oliveira R, Yang GJ, Boatin BA, Kloos H. A research agenda for helminth diseases of humans: social ecology, environmental determinants, and health systems. PLoS Negl Trop Dis. 2012;6(4):e1603 10.1371/journal.pntd.0001603 22545168 PMC3335881

[29] Closser S, Cox K, Parris TM, Landis RM, Justice J, Gopinath R, et al The impact of polio eradication on routine immunization and primary health care: A mixed-methods study. J Infect Dis. 2014;210(Suppl 1):S504–13. 10.1093/infdis/jit232 24690667 PMC4197907

[30] GHWA. Joint Commitment to Harmonized Partner Action for Community Health Workers and Frontline Health Workers. Recife, Brazil: Global Health Workforce Alliance 2013 http://www.who.int/workforcealliance/knowledge/resources/chw_outcomedocument01052014.pdf.

